# Early detection of proteinuria to prevent kidney fibrosis and progression to kidney failure: lessons from the School Urine Screening Program in Japan

**DOI:** 10.1007/s00467-025-07098-1

**Published:** 2025-12-06

**Authors:** Rini Rossanti, Kimiko Honda, Masataka Honda, Kazumoto Iijima

**Affiliations:** 1 Pediatric Nephrology Division, Department of Child Health, Faculty of Medicine, Hasan Sadikin General Hospital, Universitas Padjadjaran, Bandung, Indonesia; 2 Graduate School of Health Management, Keio University, Tokyo, Japan; 3 Department of Nephrology and Clinical Research Support Center, Tokyo Metropolitan Children’s Medical Center, Tokyo, Japan; 4 Hyogo Prefectural Kobe Children’s Hospital, Kobe, Japan; 5 Department of Advanced Pediatric Medicine, Kobe University Graduate School of Medicine, Minatojimaminami-Machi 1-6-7, Chuo-Ku, Kobe, 650-0047 Japan

**Keywords:** School urine screening, Proteinuria, Chronic kidney disease, Glomerulonephritis, IgA nephropathy, Cost-effectiveness

## Abstract

Chronic kidney disease (CKD) represents a growing global health challenge, often progressing silently until advanced stages. Persistent proteinuria plays a pivotal role in initiating the fibrotic niche—a complex cellular microenvironment contributing to early kidney fibrosis and closely associated with kidney failure. Despite advancements in CKD management, proteinuria remains underrecognized as a critical surrogate endpoint for disease progression. Urine screening for proteinuria in asymptomatic pediatric populations has been contentious due to concerns about diagnostic reliability, cost-effectiveness, and clinical relevance. However, recent data from Japan present a contrasting perspective. With a well-established nationwide school-based urine screening program, Japan has demonstrated effective early detection and intervention strategies, particularly for conditions such as IgA nephropathy. A 2024 economic evaluation reported an incremental cost-effectiveness ratio well below Japan’s pediatric willingness-to-pay threshold, affirming the program’s economic and clinical viability. These findings underscore the potential of structured screening protocols—especially in regions with underreported disease prevalence or limited healthcare access—to prevent long-term kidney impairment. Modifiable factors such as screening frequency, age of initiation, and integration of emerging biomarkers must be considered to optimize outcomes. This review highlights the need to revisit the role of proteinuria screening in pediatric nephrology, advocating for evidence-informed policy decisions that recognize its long-term value. Japan’s model offers a robust framework for balancing cost, clinical impact, and public health priorities in global efforts to address CKD in its earliest stages.

## Introduction

Chronic kidney disease (CKD) represents a growing global health burden, disproportionately affecting socially disadvantaged and marginalized populations. It imposes substantial economic costs on individuals, healthcare systems, and society at large [1]. Current estimates suggest that approximately 700 million people worldwide are living with CKD [2].

CKD often progresses silently, with significant kidney damage already present by the time clinical symptoms emerge [3]. Proteinuria is one of the earliest and most reliable indicators of kidney injury [4, 5]. Moreover, proteinuria is not merely a marker but a pathogenic contributor, triggering tubular injury, local inflammation, and activation of profibrotic pathways that ultimately lead to irreversible interstitial fibrosis and glomerulosclerosis [6, 7]. Therefore, early detection and intervention are critical to altering the trajectory of kidney disease.

Against this backdrop, Japan has pioneered a unique and systematic approach through its school urine screening (SUS) program [8]. This initiative involves annual dipstick testing for hematuria and proteinuria among nearly all students from elementary through high school. The program enables early identification of asymptomatic urinary abnormalities and timely referral to specialized care. SUS has proven effective in reducing kidney failure due to chronic glomerulonephritis (CGN) among children and adolescents, serving as a valuable tool for early detection of silent kidney disorders [9, 10]. It has facilitated early diagnosis of glomerular diseases such as IgA nephropathy, allowing for appropriate therapeutic intervention and improved long-term kidney outcomes [8, 11].

Nevertheless, the program has not been without controversy. Critics have raised concerns regarding the cost-effectiveness of large-scale screening, the psychological impact of false positives, and the potential for unnecessary interventions [12, 13]. The American Academy of Pediatrics has suggested that targeted screening of high-risk populations may offer a more balanced approach [14]. Despite these concerns, Japan’s experience provides valuable insights. When implemented systematically and coupled with a structured referral system, population-based screening can alter the natural history of pediatric kidney disease and reduce the long-term burden of CKD.

This review reexamines the role of proteinuria as an early marker of kidney fibrosis and progression to kidney failure, analyzes the epidemiological outcomes of Japan’s SUS program, and explores how these lessons may inform global strategies for kidney disease prevention. In an era of rising CKD prevalence, integrating such preventive measures into public health policy may play a pivotal role in mitigating the global burden of kidney failure.

## Proteinuria: a surrogate outcome in chronic kidney disease

Proteinuria is defined as the presence of various types of protein in the urine, including low-molecular-weight proteins (α1-microglobulin, β2-microglobulin), albumin, and high-molecular-weight proteins such as immunoglobulins G and M [15, 16]. It can be measured qualitatively using urine dipstick tests or quantitatively with the urine protein-to-creatinine ratio from a morning urine sample [17]. In the absence of a filtration barrier, approximately 26 g of albumin would enter the renal tubules per minute, while a healthy individual excretes less than 30 mg of albumin daily. Thus, healthy kidneys achieve > 99.9% efficiency in preventing albumin loss [18]. Under physiological conditions, the glomerular capillary wall effectively restricts protein passage into Bowman’s space by considering molecular size, electric charge, and steric structure [16].

**Fig. 1:**
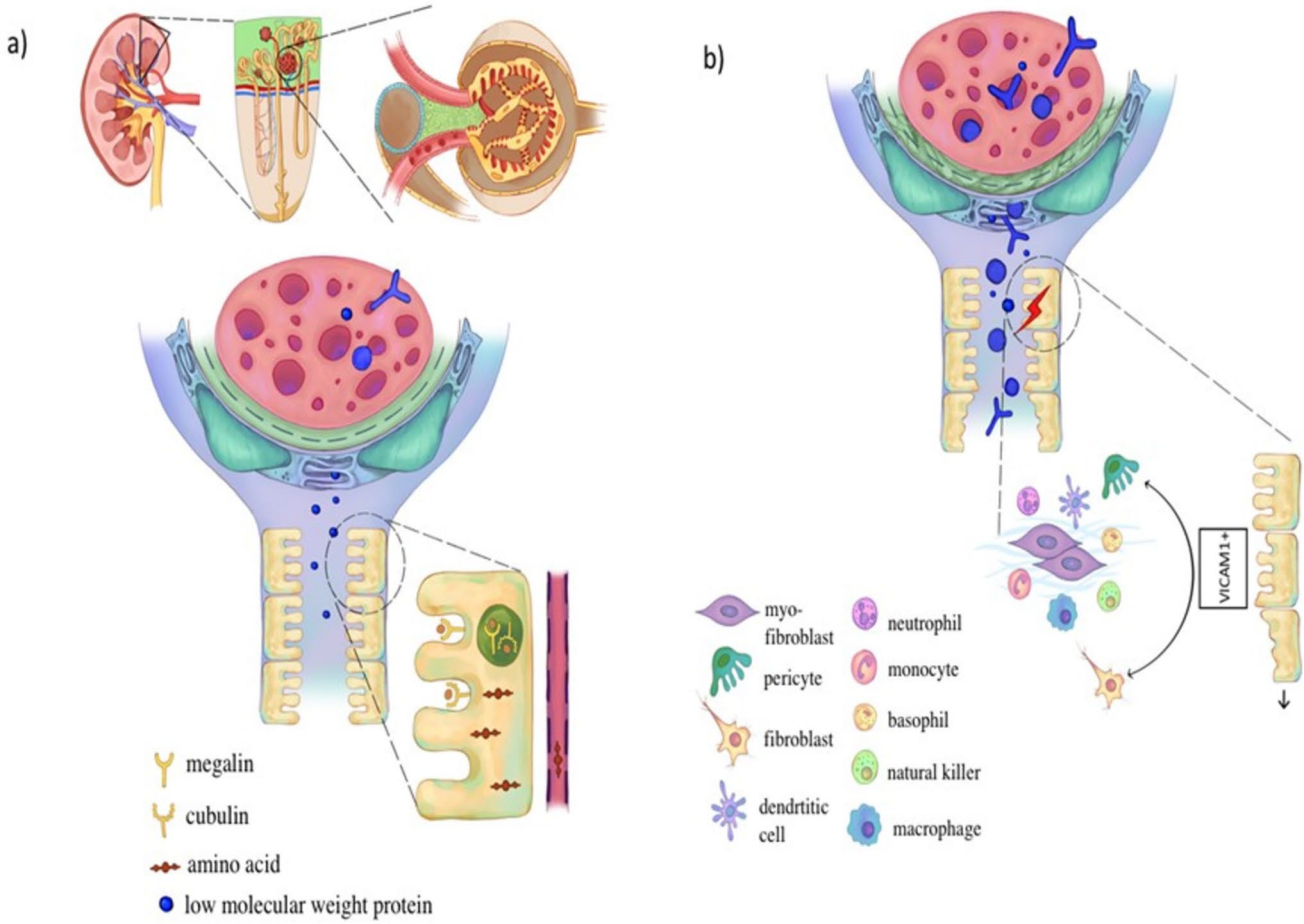
Schematic illustration of glomerular protein filtration. **a** Protein filtration under physiological conditions. The slit diaphragm, approximately 40 nm in length with pore sizes ranging from 50 to 150 Å, permits the passage of small proteins (blue dots) into the urinary space. Under normal physiological conditions, nearly all of these proteins that enter the tubular lumen are reabsorbed by proximal tubule epithelial cells, resulting in only trace amounts appearing in the final urine. This reabsorption is mediated by the apical endocytic receptors cubilin and megalin. Once internalized, proteins are degraded within lysosomes into amino acids, which are subsequently released into the bloodstream via the basolateral membrane. **b** Excessive proteinuria induces a ‘fibrotic niche’. In cases of severe or chronic proteinuria, intracellular systems such as the endoplasmic reticulum and lysosomes—responsible for processing reabsorbed albumin—become overwhelmed or dysfunctional. Injured VCAM-1⁺ tubules influence interstitial pericytes and fibroblasts, promoting myofibroblast differentiation, proliferation, and extracellular matrix accumulation. Notably, distinct immune cell populations—including neutrophils, basophils, lymphocytes, and macrophages—are also observed in the interstitial space. These immune cells play a critical role in the expression of fibrosis-associated markers and contribute to the progression of kidney fibrosis

Proteinuria is a key indicator of kidney dysfunction, reflecting abnormalities in the glomerular filtration barrier [15]. If left untreated, it can lead to kidney fibrosis and eventually progress to kidney failure. Normally, the tubular epithelium reabsorbs excess protein that reaches the tubules. However, when this system becomes overloaded, it activates inflammatory and fibrotic pathways, contributing to glomerulosclerosis and interstitial fibrosis [19] (Fig. 1). Interestingly, proteinuria-induced tubular damage may exacerbate glomerular pathology, potentially creating a vicious cycle. Selective tubular injury alone has been implicated in the development of glomerulosclerosis and proteinuria [20, 21].

**Fig. 2:**
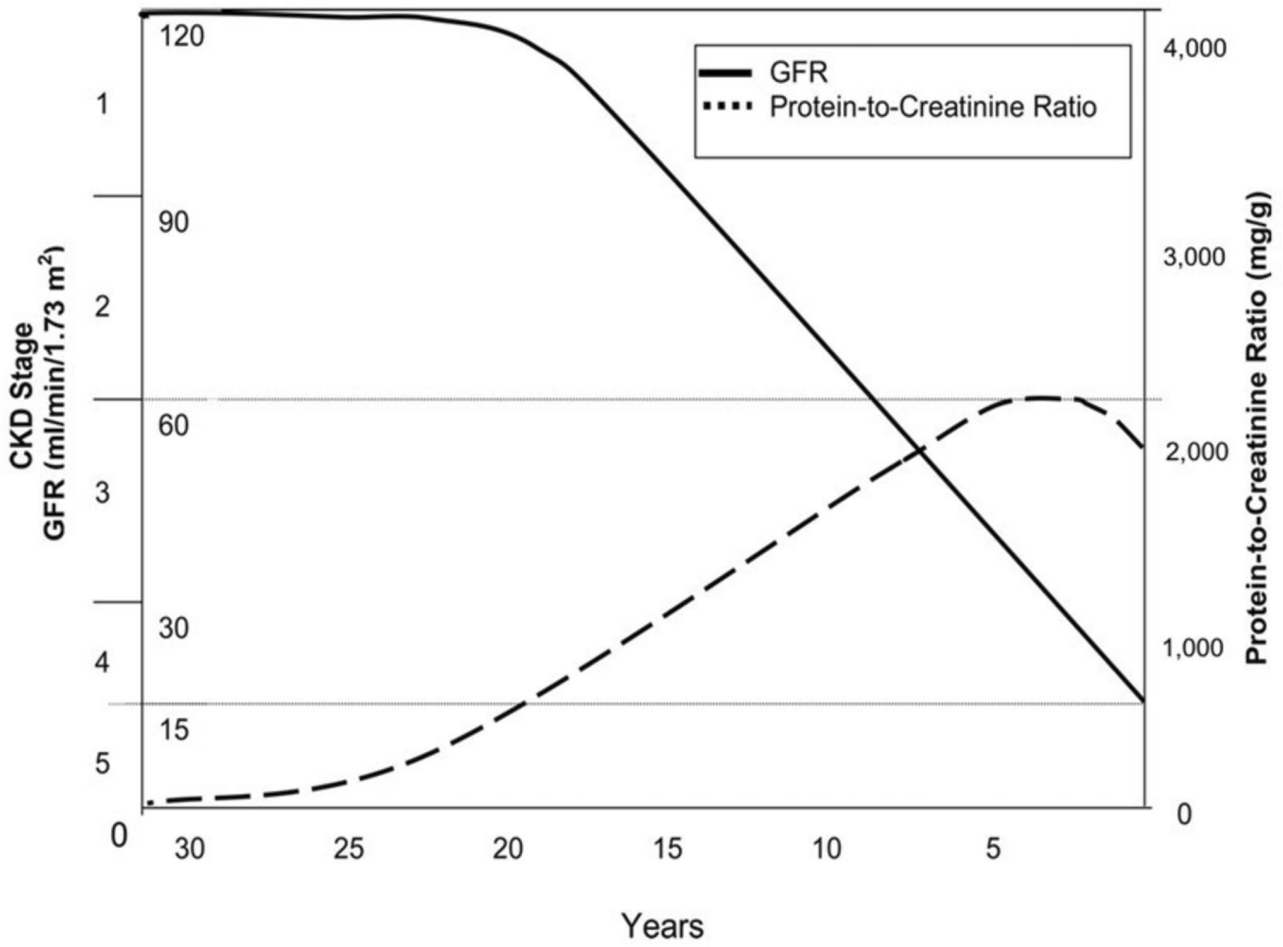
The progression of kidney disease by showing changes in GFR and proteinuria over time. Proteinuria is the initial sign of kidney disease, and it increases early on and is elevated for the duration of the illness. After about 15 years, GFR starts to decline and eventually reaches levels linked to kidney failure after 30 years (under permission by CJASN, Surrogate End Points for Clinical Trials of Kidney Disease Progression 1(4):874–884, July 2006, adapted from Fig. 2) [5]

To assess and monitor the progression of CKD, it is crucial to identify valid surrogate endpoints that reflect treatment effects on clinical outcomes. Biomarkers indicating treatment-induced changes in CKD progression are essential [5]. A recent meta-analysis demonstrated that proteinuria serves as a reliable surrogate endpoint for CKD progression, replacing glomerular filtration rate (GFR) < 15 ml/min per 1.73 m^2^ as a distal, less accessible marker with a more proximal and measurable indicator [1]. Evidence suggests a strong correlation between estimated GFR (eGFR) decline and proteinuria severity. These findings highlight the importance of early proteinuria detection and treatment to mitigate CKD progression [5] (Fig. 2).

## Preventing the kidney fibrotic niche: a proteinuria perspective

The fibrotic niche—characterized by complex cell–cell interactions initiated by severe or persistent proteinuria—is closely associated with kidney failure [22, 23]. Kidney fibrosis, the final common pathway in CKD, ultimately leads to kidney failure, incurring substantial personal, healthcare, and societal costs [6, 24]. CKD typically progresses slowly and asymptomatically, regardless of the underlying etiology or rate of kidney injury [22]. While kidney failure (defined as GFR < 15 ml/min per 1.73 m^2^ or the need for dialysis/transplantation) serves as a recognized clinical endpoint for CKD studies [1], its measurement is often unfeasible due to the slow disease course. Long-term follow-up is required, and many patients die of cardiovascular or CKD-related complications before reaching kidney failure [5].

In healthy individuals, total urinary protein excretion should not exceed 150 mg/day, and albuminuria should remain below 26 mg/day [18]. In clinical practice, albuminuria at a level of < 30 mg/g Cr is considered normal, whereas levels between 30 and 300 mg/g Cr are considered moderately increased and levels > 300 mg/g Cr are considered severely increased [25]. Semiquantitative dipstick results are classified as follows: 1 + (30 to < 100 mg/dL), 2 + (100 to < 300 mg/dL), 3 + (300 to < 1000 mg/dL), and 4 + (≥ 1000 mg/dL) [25]. Proteinuria is often detected through routine laboratory testing and may be asymptomatic [15]. As proteinuria worsens, hypoalbuminemia decreases plasma colloid osmotic pressure, enhancing transcapillary water filtration and gravitational fluid shift [26].

**Fig. 3:**
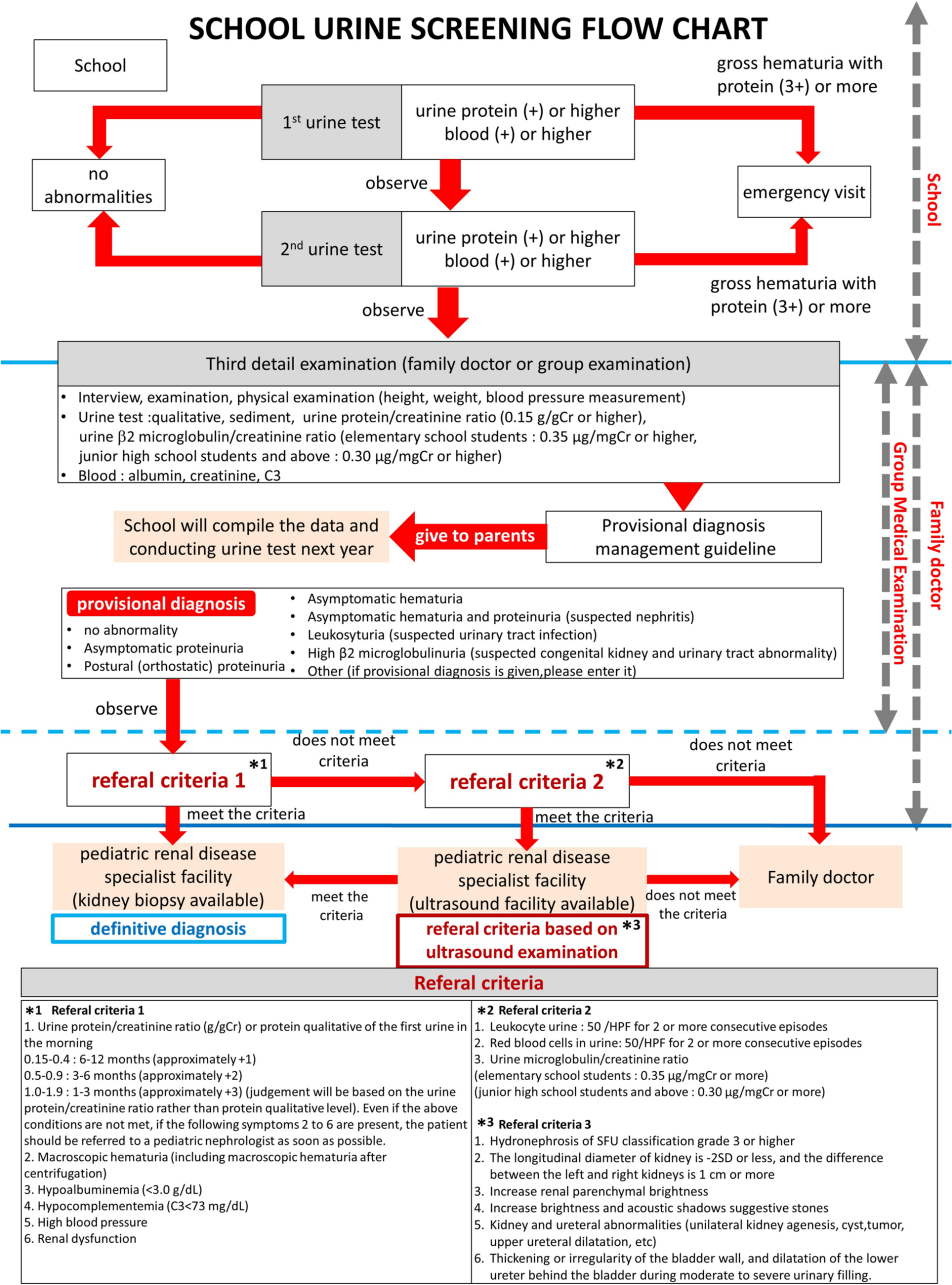
School urine screeningflow chart in Japan. Adapted from SCHOOL URINE SCREENING FLOW CHART in the Manual for Pediatric Urine Screening (in Japanese), Second edition, edited by the Japanese Society for Pediatric Nephrology, 2022, Shindan to Chiryo Sha, Inc., Tokyo (under permission by Shindan to Chiryo Sha, Inc., Inc.)

Early manifestations of proteinuria include periorbital and peripheral edema. Over time, fluid retention increases, resulting in weight gain and potential development of pleural effusions or ascites [27]. Massive proteinuria is defined as ≥ 1000 mg/m^2^/day in a 24-h urine sample, corresponding to 3 + or 4 + results by dipstick [28]. At this stage, urine screening plays a vital role in both primary prevention, by identifying individuals before any signs of kidney damage appear, and secondary prevention, by detecting and managing early kidney disease to prevent its progression. Proactive monitoring enables early intervention, delays CKD progression, and helps preserve kidney function. Japan’s SUS program exemplifies comprehensive early detection efforts (Fig. 3). At the tertiary prevention level, a reduction in proteinuria to < 1 g/24 h within the first year of diagnosis strongly predicts long-term kidney function preservation—surpassing baseline proteinuria or GFR as a prognostic factor [29].

## SUS for asymptomatic proteinuria and hematuria in Japan and other countries

SUS for asymptomatic proteinuria and hematuria in Japan was launched in 1974 by the Ministry of Education, Culture, Sports, Science and Technology, following the enactment of the School Health Law in 1973. At that time, kidney disease was the leading cause of long-term health-related absenteeism, and early diagnosis and management were considered essential for reducing school truancy. Indeed, SUS in Japan proved highly successful in detecting asymptomatic kidney disease at an early stage [8].

Figure 3 illustrates the SUS flow chart implemented in Japan. Each year, school teachers provide students with detailed instructions on how to properly collect a first-morning urine sample. Following these guidelines, students bring a first-morning urine sample collected at home to school, where nongovernmental laboratories conduct tests in cooperation with school physicians, nurses, and teachers. Each sample is dipstick-tested, with a cutoff of (+) for both proteinuria and occult blood. If proteinuria reaches (3 +), parents are notified by phone or mail. In the case of positive test results, the student is asked to test the second urine sample about 4 weeks later. If the test remains positive, a printed form is provided to parents with instructions for follow-up examinations, which may be conducted via mass screening or by school physicians, family physicians, or public health centers. These follow-ups include medical history review, urinalysis, physical examination, blood pressure measurement, urine protein-to-creatinine ratio (uPCR), beta2-microglobulin-to-creatinine ratio, and blood tests such as albumin, creatinine, and complement levels. After the detailed examination, the attending physician or an evaluation committee makes a provisional diagnosis. Children are instructed to urinate before going to bed on the night before the examination. However, if they do not follow this instruction and fail to urinate before bedtime, or if they are physically active before sleep, the effects of upright posture and mild physical activity may be reflected in the urine sample, potentially resulting in the detection of proteinuria. As a result, orthostatic proteinuria can be included as a provisional diagnosis in the screening flowchart. All students who receive follow-up examinations are required to submit the results to their school.

Referral to pediatric nephrologists is primarily based on uPCR findings and proteinuria levels on dipstick. Additionally, cases exhibiting gross hematuria, hypoalbuminemia, decreased serum complement levels, hypertension, or impaired kidney function warrant prompt referral. These designated facilities, recognized by the Japanese Society for Pediatric Nephrology, are staffed by pediatric nephrologists equipped to perform kidney biopsies and manage glomerulonephritis and other forms of chronic kidney disease [30].

In Chiba City, between 1975 and 2011, 75,000 to 129,000 students (98% of all students) underwent annual SUS. The positive rate for primary urine screening was 1.65–2.30%, with 0.35–0.68% positivity in secondary screening and 0.22–0.43% in detailed examinations. Among 9544 students who underwent detailed examinations, 334 (3.5%) were diagnosed with kidney diseases; 310 (3.2%) had chronic glomerulonephritis (CGN). Of these, 204 (2.1%, 65.8% of CGN cases) were diagnosed with IgA nephropathy, 54 with non-IgA proliferative glomerulonephritis, 22 with membranoproliferative glomerulonephritis, 15 with membranous nephropathy, and 4 with focal segmental glomerulosclerosis [30, 31].

In 1990, a multicenter clinical trial was initiated to evaluate the efficacy and safety of combination therapy with steroids and immunosuppressants for children with newly diagnosed severe IgA nephropathy, a condition known to carry a poor kidney prognosis. Of note, in this trial, 82% of enrolled patients had asymptomatic proteinuria and hematuria identified through SUS, allowing for early diagnosis and early treatment. After 2 years, patients receiving combination therapy showed significantly reduced proteinuria and histological improvements. In contrast, those treated solely with antiplatelet and anticoagulant agents showed no improvement in proteinuria and experienced significantly increased glomerulosclerosis [32]. Long-term outcomes from this study demonstrated significantly higher cumulative kidney survival in the combination therapy group compared to controls (10-year survival 97.1% vs. 84.8%, log-rank *p* = 0.03) [11].

By the early 1990 s, angiotensin converting enzyme (ACE) inhibitor therapy for IgA nephropathy had become standard practice worldwide [33]. In Japan, following the above trial, steroid- and immunosuppressant-based therapies for newly diagnosed severe IgA nephropathy became widespread [34–36]. For patients with mild to moderate disease, treatment with ACE inhibitors or angiotensin II receptor blockers (ARBs) was also actively implemented [37, 38]. Yata et al. retrospectively assessed the long-term prognosis of 500 pediatric-onset IgA nephropathy cases in Japan. Among those diagnosed between 1976 and 1989 (*n* = 219), 10-, 15-, and 20-year kidney survival rates were 94.0%, 80.1%, and 70.1%, respectively. For those diagnosed between 1990 and 2004 (*n* = 281), survival rates were 98.8% at both 10 and 15 years [39].

Yamagata et al. analyzed kidney replacement therapy registries from 1983 to 1999 in Japan and the USA. They found that in patients under 45 years with kidney failure due to glomerulonephritis, incidence decreased in the Japanese cohort exposed to SUS, while no similar trend was observed in the US population [40]. Harambat et al. reported that although Japan has one of the highest adult kidney failure rates globally, the pediatric kidney failure rate is notably low at 4.3 per million age-related population (pmarp), compared to 9.5 in 11 Western European countries and Australia, and 15.5 in the USA [41]. According to 1998 registry data on pediatric kidney failure in Japan, the proportion attributable to glomerulonephritis (excluding focal segmental glomerulosclerosis) was 13.3%, lower than the 19.6% observed in the USA between 1993 and 1997 [9]. From 2006 to 2011, this rate declined further to 5.9% in Japan, while in the USA, it remained at 10.1% in 2022 [10].

In Taiwan, SUS has been conducted semiannually among public and private elementary and junior high school students since 1990. In South Korea, SUS has been implemented among students at all school levels since 1998. The prevalence of positive proteinuria results in two early morning urine tests is approximately 0.4% in both countries, closely mirroring rates in Japan [42, 43].

In Taiwan, during SUS implementation between 1992 and 1996, the proportion of children undergoing dialysis due to glomerulonephritis fell from 63.2% to 47.0% [44]. According to data from the Korean Society of Nephrology, the proportion of new kidney failure cases caused by CGN fell from 25.3% in 1992 to 11.1% in 2009 [45].

The decline in the proportion of kidney failure attributable to GN in the USA is less evident compared to the reductions reported in countries like Japan that have implemented SUS programs. This differential trend aligns with the hypothesized benefit of earlier (upstream) detection and management of GN facilitated by SUS settings. However, as incident kidney failure reflects multiple system-level determinants, therapeutic advances, and environmental factors, it is difficult to quantitatively ascertain the specific contribution of SUS to these cross-sectional differences.

Nevertheless, evidence from Japan, particularly concerning pediatric IgA nephropathy identified through SUS, supports the view that earlier case ascertainment and timely pharmacological intervention (renin–angiotensin system blockade and immunosuppression) can modify disease trajectories and improve long-term kidney outcomes. This perspective is consistent with the pediatric literature outside Japan, which also argues for the importance of early diagnosis and treatment of IgA nephropathy in childhood [46]. While definitive causal confirmation remains challenging, these observations may be interpreted as consistent with a potential contribution of SUS, via earlier detection of glomerulonephritis including IgA nephropathy, to reductions in glomerulonephritis-related kidney failure.

In contrast, although the following policy was retired in June 2025, the American Academy of Pediatrics removed routine urinalysis from its 2007 health supervision guidelines [47], citing concerns over frequent false-positive results, increased costs, and unnecessary invasive procedures with limited diagnostic benefit [14]. Dodge et al. reported a high proteinuria positivity rate in US children (11.7%), often due to transient or orthostatic proteinuria [48]. In their study, proteinuria (> 10 mg/dL) was initially detected in 1440 children, yet only 736 (51.1%) remained positive on follow-up samples within 2 to 7 days. Moreover, only 2.1–3.2% tested positive on all three specimens [48]. Even these final rates remain far above the 0.4% observed in SUS programs in Japan, Taiwan, and South Korea. A primary reason for this discrepancy may lie in differences in sampling methods: Dodge’s study utilized spot urine samples, whereas SUS programs rely on early morning urine. Supporting this interpretation, 61.8% of positive cases in the US study were diagnosed with orthostatic proteinuria, a condition more likely to be detected in spot samples. In addition, differences in the definition of proteinuria thresholds may contribute; Dodge’s study used a cutoff of 10 mg/dL, while the SUS programs define proteinuria as a semiquantitative dipstick result of 1 + or higher, which corresponds to ≥ 30 mg/dL. Hogg noted a lack of international consensus on whether CKD screening should be performed in children and adolescents [12]. While SUS remains well established in Japan, Taiwan, and South Korea, there has been a shift away from routine pediatric CKD screening in North America and Europe due to cost-effectiveness concerns [12].

## Cost-effectiveness of urine screening in asymptomatic youths and its potential applicability across different countries

The cost-effectiveness of urine screening among asymptomatic youths has long been a subject of debate, primarily due to concerns regarding false-positive results and associated economic burden [12]. High rates of transient abnormalities and false positives raise issues around unnecessary follow-up evaluations and increased anxiety among families [13]. To date, only two economic evaluations of urine screening in asymptomatic youths have been conducted—by Kaplan et al. [49] and Sekhar et al. [50]—both concluding that screening was not cost-effective. However, these studies did not consider the clinical benefits of early detection and intervention, despite CKD having a profound impact on patient quality of life and social outcomes, with annual dialysis costs reaching $66,000 per patient [51].

In 2024, Honda et al. conducted an economic evaluation of school-based urine screening in Japan, focusing on its value for earlier detection and treatment of IgA nephropathy, while explicitly accounting for false-positive burden [52]. To our knowledge, this is the first comprehensive economic evaluation of universal CKD screening in asymptomatic children. Their analysis found that this screening strategy was effective in reducing the number of patients developing kidney failure due to IgA nephropathy. The incremental cost-effectiveness ratio (ICER) was 4.18 million JPY per QALY—well below Japan’s willingness-to-pay threshold of 7.5 million JPY per QALY for pediatric diseases—indicating the cost-effectiveness of the screening program.

These results are not automatically generalizable. Cost-effectiveness must be assessed in each country’s context—health-system design, unit costs and budgets, epidemiology, and clinical practice—and the current evidence consists only of this Japanese evaluation. Nevertheless, these findings may inform decisions on whether and how to implement pediatric CKD screening elsewhere. Sensitivity analyses identified the key value drivers. Excluding the discount rate, the largest effects on the ICER came from (i) system/process costs (unit costs for screening and confirmatory testing, false-positive rate); (ii) epidemiology (incidence of IgA nephropathy, the probability of incidental detection without screening, the probability that severe IgA nephropathy progresses to kidney failure without treatment, and the proportion of severe disease); and (iii) the probability that IgA nephropathy not detected during childhood progresses to kidney failure in adulthood (after age 18). By contrast, cross-country differences in practice patterns, including pediatric biopsy rate and treatment-response probability, had relatively small effects and were less consequential for cost-effectiveness than differences in epidemiology and costs.

These findings imply that, in regions where IgA nephropathy incidence is an order of magnitude lower than in Japan (e.g., much of Europe and North America), IgA nephropathy-focused strategies are unlikely to be cost-effective—even allowing for possible under-ascertainment without screening and differences in biopsy practice. Moreover, cost-effectiveness is also highly sensitive to program costs; if new logistics must be developed, many years may be needed to amortize fixed costs. Therefore, copying and pasting Japanese programs is seldom optimal.

On the other hand, CKD carries a substantial global burden; in resource-limited areas, many people with kidney failure have limited access to kidney-replacement therapy, and the costs can be financially catastrophic even when kidney-replacement therapy is available [24, 53]. Historically, early detection received less emphasis because outcomes in pediatric CKD were viewed as only marginally modifiable. However, evidence now shows that proteinuria predicts faster progression and that renin–angiotensin–aldosterone system inhibition slows decline in children, while long established in adults [54–56]. Thus, whether glomerular or non-glomerular in origin, earlier identification offers a chance to modify trajectories. As mentioned above, SUS detects not only IgA nephropathy but also other nephritides and CAKUT, yet Honda et al.’s cost-effectiveness analysis does not include the benefits of detecting these conditions.

Given this broader benefit, countries can design context-appropriate screening: if the goal is early detection of proteinuria, hematuria testing is not essential; with appropriate training, school staff or primary-care teams can confirm dipstick proteinuria without outsourcing, and implementation need not be school-based. Where budgets are constrained, as illustrated by Honda et al.’s scenario analyses [52], costs can be reduced by raising the starting age and/or lengthening screening intervals. Conversely, more sensitive quantitative tests such as the uPCR or albumin-to-creatinine ratio (ACR) may be preferable to dipstick protein in settings prioritizing diagnostic accuracy or targeting high-risk populations. Because reagent strips measure concentration, low specific gravity can reduce sensitivity, whereas uPCR and ACR normalize to creatinine and mitigate dilution effects—an advantage particularly relevant in children with congenital anomalies of the kidney and urinary tract, who may exhibit impaired urine concentrating ability. Nevertheless, the higher unit costs of ACR and uPCR compared with dipstick urinalysis may limit their feasibility for universal screening, although their use could be considered depending on available resources and program priorities. Sekhar et al. previously concluded that well-child urinalysis was not cost-effective; nevertheless, they cautioned that “the cost-effectiveness of this procedure may change if the benefits of early treatment alter outcomes” [50]. With pediatric evidence now indicating modifiable trajectories, a context-specific reappraisal of screening is warranted.

## Conclusions

Urine screening in asymptomatic youths remains a contentious issue, yet recent findings suggest its strategic value in early detection and intervention, particularly for IgA nephropathy. In Japan, compared to Western countries, there is a tendency to perform kidney biopsies even in cases of milder urinary abnormalities. For example, in cases of isolated proteinuria, a kidney biopsy is considered appropriate if transient or orthostatic proteinuria and low-molecular-weight proteinuria have been ruled out, and if an early morning uPCR ≥ 0.5 g/g Cr persists for more than 3 months. It is well known that any glomerular disease may present with mild to moderate proteinuria as its only manifestation [57]. Therefore, we believe it is worth reconsidering the possibility that early diagnosis through kidney biopsy—undertaken with careful attention to safety—may lead to timely and appropriate treatment, ultimately helping to prevent progression to kidney failure.

Although prior economic evaluations have questioned cost-effectiveness due to high false-positive rates and transient findings, these analyses often excluded long-term clinical benefits and broader societal impact. Emerging data from Japan indicate that school-based urine screening can significantly reduce progression to kidney failure, with an ICER below national thresholds for pediatric cost-effectiveness.

This approach may be particularly advantageous in settings where disease prevalence is underrecognized and healthcare access is limited. Moreover, policy modifications—such as adjusting screening age or frequency—may optimize cost–benefit ratios. Continued refinement of screening methodologies, including the integration of novel biomarkers and long-term efficacy data from emerging therapies, is crucial for maintaining relevance.

Taken together, the evidence supports revisiting the role of urine screening in preventive nephrology. While generalizability requires context-specific evaluation, the Japanese model offers a compelling framework for other nations seeking to enhance early detection strategies within pediatric populations.

## Data Availability

Not applicable.
